# Perception of hypertension and adherence to hypertension treatment among patients attending a hospital in western Iran: A cross‐sectional study

**DOI:** 10.1002/hsr2.1501

**Published:** 2023-08-17

**Authors:** Parastoo Baharvand, Farideh Malekshahi, Amirpourya Babakhani

**Affiliations:** ^1^ Department of Social Medicine Social Determinants of Health Research Center, School of Medicine, Lorestan University of Medical Sciences Khorramabad Iran; ^2^ Department of Social Medicine School of Medicine, Lorestan University of Medical Sciences Khorramabad Iran

**Keywords:** hypertension, medication adherence, perception, treatment adherence

## Abstract

**Background and Aims:**

Hypertension is the third leading cause of death in the world and is estimated to be increased by about 60% by 2025. Beliefs about hypertension can predict patient adherence to hypertension treatment. This study aims to investigate the perceptions of hypertension and adherence to hypertension treatment among patients in Khorramabad, Iran.

**Methods:**

This is a descriptive/analytical study with a cross‐sectional design. Participants were 265 patients with a history of hypertension referred to a hospital in Khorramabad, Lorestan Province in western Iran in 2020, who were selected using a convenience sampling method. A demographic form, the brief illness perception questionnaire‐revised (BIPQ‐R), and Morisky medication adherence scale (MMAS‐8) were used for collecting data. The collected data were analyzed in SPSS v.22 software using descriptive statistics, Pearson's correlation test, independent *t*‐test, one‐way ANOVA, and regression analysis.

**Results:**

The mean scores of BIPQ‐R and MMAS‐8 were 49.05 ± 15.45 (out of 80) and 3.69 ± 1.62 (out of 8), respectively. There was a significant relationship between the mean scores of MMAS‐8 and BIPQ‐R in total (*p* < 0.001). Perceptions of illness consequences (*B* = 4.59, *p* = 0.005), personal control (*B* = 0.190, *p* = 0.047), and symptoms (*B* = 1.77, *p* = 0.005) could significantly predict treatment adherence of patients. In illness perception, there were significant differences among patients with different places of residence (*p* = 0.032), educational levels (*p* = 0.001), and employment status (*p* = 0.010). In treatment adherence, there were significant differences among patients with different places of residence (*p* = 0.042) and educational levels (*p* = 0.045).

**Conclusion:**

Treatment adherence of hypertensive patients in western Iran is at a low level, while their perception of hypertension is at a moderate level. Clinical physicians are recommended to pay attention to the perception of illness in these patients (especially unemployed and less educated patients living in rural areas) to improve their adherence to treatment and blood pressure control.

## 1 INTRODUCTION

Nowadays, high blood pressure or hypertension has become a growing problem. It is the most common cardiovascular disease.^1^ It is the third leading cause of death in the world and is expected to cause 7.5 million deaths, accounting for about 12.8% of the total deaths per year.^2^ About two‐third of the population with hypertension globally live in low‐ and middle‐income countries.^3^ Studies have shown that the prevalence of hypertension in Iran is 25%.^4^, ^5^ By 2025, the number of people with hypertension is estimated to increase by about 60%, reaching 1.5 billion.^6^ Therefore, to improve prognosis, early detection and treatment of hypertension are necessary. Globally, almost 31% of the adult population is affected by hypertension, among which only 36.9% go under treatment and 13.8% having can control their hypertension.^7^ Treatment of hypertension and control of blood pressure can decrease cardiovascular events, morbidity, and mortality in both men and women.^8^ Pharmacological and nonpharmacological methods are used in the treatment of hypertension. The main nonpharmacological measures are lifestyle modifications such as dietary regime, exercise, avoiding stress, or reducing alcohol consumption.^9^, ^10^ Adherence to treatment and medication is important to control high blood pressure. Low adherence to treatment is a major barrier to blood pressure reduction and control.^11^ Lack of or poor treatment adherence increases cardiovascular morbidity and mortality.^12^, ^13^, ^14^ According to the World Health Organization, treatment adherence is “the extent to which a person's behavior—taking medication, following a diet, and/or executing lifestyle changes—corresponds with the agreed recommendations from a healthcare provider.”^15^ Beliefs about specific medications and perception of illness are predictive of patient adherence to antihypertensive treatment.^16^ Patients may not continue with treatment if they perceive it poorly.^17^ Therefore, having knowledge of patient's perceptions are necessary for improving their adherence to treatment. Negative perceptions of illness are associated with more delay in recovery process and an increase in healthcare use; these perceptions can be modified by providing appropriate education.^18^

Few studies have been conducted to assess both treatment adherence and perception of illness in hypertensive patients. Hsiao et al. in a study in 2012 on hypertensive patients referred to the family physician clinic of a medical center in Taiwan, investigated the perceptions of illness in hypertensive patients and their associations with medication adherence. Their results showed that the patients perceived their hypertension as a chronically severe but stable disease and were sure about the efficacy of medical treatments and were able to control their disease. They recommended that clinical physicians should pay attention to patients' illness perceptions, including their negative emotional responses and symptoms to improve their adherence to medication.^19^ Saarti et al. in a study in 2016 examined medication adherence and illness perception in hypertensive patients in Lebanon. Based on their results, 29.1% had low treatment adherence, and the likelihood of hypertension control in patients with high adherence was 3.5 times higher than in patients with low adherence. They found no significant difference in illness perception between adherent and nonadherent patients.^20^ Taheri‐Kharameh et al. evaluated illness perception and adherence to treatment among patients with hypertension referred to teaching hospitals in northern Iran (Qom City) in 2016. About 35% of patients showed higher adherence to treatment. Personal control and identity of symptoms were associated with more adherence.^21^ Doust Mohammadi et al. in 2018 assessed illness perception and adherence to treatment among hypertensive patients in Tehran, Iran, and found a significant association between them.^22^ Guimarães et al. in 2016 assessed Portuguese patients' hypertension perceptions and the relationship between their perceptions and adherence to medication. Patients reported a high level of medication adherence and a high frequency of adherence behaviors, where 20.6% stated that they did not take drugs because they forgot it.^23^ Shakya et al. assessed illness perception and treatment adherence among hypertensive patients referred to a tertiary hospital in Nepal in 2020. Most of them perceived hypertension as a highly threatening illness and as a chronic disease, and had a high level of personal and treatment control. They found a significant positive relationship between the perception of illness and adherence to treatment.^18^ Otenyo and Kereri investigated the influence of illness perception on adherence to medication in patients with hypertension admitted to a hospital in Kenya in 2021. They found that 33.3% of the patients had a high adherence level.^24^ Rashid et al. assessed the perception of illness and adherence to medication in hypertensive patients in Bangladesh in 2020 and reported their very low level of adherence. According to their findings, 63% of patients perceived that hypertension is not curable. Furthermore, 94% missed their follow‐up, despite that their physician asked them for follow‐up.^25^

Treatment adherence is essential for optimal blood pressure control, and understanding patients' perceptions of illness are essential for improving treatment adherence. In this regard, and considering that no study was found on surveying both hypertension treatment adherence and perception of hypertension in western Iran, the current study aims to investigate hypertension treatment adherence and perception of hypertension in patients referred to a teaching hospital in western Iran. We also evaluated the association between their hypertension treatment adherence and perception of hypertension.

## 2 METHODS

This is a descriptive/analytical study with a cross‐sectional design, approved by the ethics committee of Lorestan University of Medical Sciences, Lorestan, Iran (Code: IR.LUMS.REC.1399.223). The study population consists of all patients with a history of hypertension stage II (systolic blood pressure at least 140 mmHg or diastolic blood pressure at least 90 mmHg) referred to the cardiovascular clinic of Madani Hospital in Lorestan, Iran in 2020 (during the COVID‐19 pandemic). Using the formula: *n* = (Z_α_ ×  Z_β_)^2^/(ω)^2^ + 3 where ω = 1/2 In([1+r]/[1−r]), *α* = 0.05, *β* = 0.20, and *r* = 0.181 according to a previous study,^22^ the sample size was determined 265. In this regard, 265 eligible patients were selected using a convenience sampling technique. Inclusion criteria were willingness to participate in the study, a definite diagnosis of hypertension by a cardiologist, having hypertension for at least 6 months, reading and writing literacy, no mental or cognitive disease, and taking medication due to primary hypertension. Having other chronic diseases (e.g., diabetes), unwillingness to continue participation, or returning incomplete questionnaires were the criteria for exclusion from the study. Informed consent was obtained from all individual participants included in the study.

Data collection tools were a demographic form (surveying age, gender, occupation, education, marital status, and place of residence), the brief illness perception questionnaire–revised (BIPQ‐R), and the Morisky medication adherence scale (MMAS‐8). The BIPQ‐R is a 9‐item scale designed by Broadbent et al.^26^ to assess illness perceptions. It has 9 items, where items 1−8 are rated on a scale from 0 to 10 assessing illness consequences, illness duration, personal control, treatment control, identity (symptoms), illness control, understanding, and emotional response, and item 9 is an open‐ended question assessing causal perceptions by asking patients to list the three most likely causes for their illness. The total score ranges from 0 to 80. Higher scores indicate stronger perceptions about that dimension. Broadbent et al. reported a good test−retest reliability and good concurrent, predictive, and discriminant validity of BIPQ‐R. We used the Persian version of BIPQ‐R validated by Masaeli et al.^27^ They showed its acceptable reliability (*α* = 0.59−0.73).

The MMAS‐8 is a structured self‐report measure of medication‐taking behavior developed by Morisky et al.^28^ This measure was designed to facilitate the recognition of barriers to and behaviors associated with adherence to chronic medications. The scale provides information on behaviors related to medication use that may be intentional (e.g., not taking medications because of side effects) or unintentional (e.g., forgetting to take medication). It has 8 items. Response choices are “yes” (0 points) or “no” (1 point) for items 1−7 except for the item 5, where “yes” has 1 point and “no” has 0 points. The scoring for the item 5 is reversed. Item 8 has a 4‐point Likert response scale (0 = *never/rarely*, 0 = *sometimes*, 1 = *usually*, 1 = *always*). The MMAS‐8 was originally used in hypertensive patients and the results revealed that it was a reliable tool (*α* = 0.83) and had a significant correlation with blood pressure control. It showed a sensitivity of 93% in detecting patients with poor blood pressure control.^29^ We used the Persian version of MMAS‐8 validated by Moharamzad et al.^30^ for hypertensive patients in Iran. They showed its acceptable internal consistency (*α* = 0.697), and good test−retest reliability (*r* = 0.94). Total score ranges from 0 to 8, where a score of 8 shows high adherence, a score of 6−7 indicates moderate adherence, and a score <6 shows low adherence.

After obtaining ethical approval from the university and obtaining informed consent from the participants, questionnaires were distributed among them in the clinic by the last author. After their completion, their data were analyzed in SPSS v.22 software using descriptive statistics (mean, standard deviation, frequency, percentage), Pearson's correlation test (to examine the relationship between the scores of MMAS and BIPQ‐R), independent *t*‐test (to examine the difference in MMAS‐8 and BIPQ‐R scores of patients in terms of age, gender, and place of residence), one‐way ANOVA (to examine the difference in MMAS‐8 and BIPQ‐R scores of patients in terms of marital status, occupation, and education), and multiple regression analysis (to find the predictors of treatment adherence). The results of Kolmogorov−Smirnov test showed that the data had normal distribution for both study variables (*p* > 0.05). The significance level was set at 0.05.

## 3 RESULTS

Of 265 participants, 100 were male (37.7%) and 165 female (62.3%). The mean age of participants was 48 ± 13.26 years ranged from 19 to 88 years. Most of participants were married (*n* = 151, 57%) and self‐employed (*n* = 86, 32.5%), with an academic degree (*n* = 106, 40%), living in urban areas (*n* = 208, 78.5%). For more information, see Table 1.

**Table 1:** Demographic characteristics of participants (*n* = 265).

Characteristics		*N* (%)
Gender	Male	100 (37.7)
	Female	165 (62.3)
Marital status	Single	82 (30.9)
	Married	151 (57)
	Divorced	32 (12.1)
Age (year)	≤40	102 (38.5)
	>40	163 (61.5)
Educational level	Lower than high school	78 (29.4)
	High school diploma	81 (30.6)
	Academic degree	106 (40)
Occupation	Self‐employed	86 (32.5)
	Employed	51 (19.2)
	Housekeeper	50 (18.9)
	Unemployed	78 (29.4)
Place of residence	Urban areas	208 (78.5)
	Rural areas	57 (21.5)

Based on the answers to the questions in BIPQ‐R, most of patients perceived that: the illness had “moderately” affected their life (rated 5 out of 10; *n* = 61, 23%); their illness would continue “for a while” (rated 4 out of 10; *n* = 43, 16.2%); they had a “moderate” amount of control over their illness (rated 6 out of 10; *n* = 45, 17%); treatment was “moderately” helpful for them (rated 5 out of 10; *n* = 62, 23.4%); they experienced some symptoms from their illness (rated 5 out of 10; *n* = 59, 22.3%); they were “moderately” concerned about their illness (rated 4 out of 10; *n* = 66, 24.9%); they understood their illness “moderately” (rated 4 out of 10; *n* = 47, 17.7%); and their illness “moderately” affected them emotionally (rated 5 out of 10; *n* = 68, 25.7%). Regarding the item 9 stating “Please list the three most important factors that you believe caused your hypertension,” 45% mentioned “inactivity” and the rest left it blank. The mean total score of BIPQ‐R was obtained 49.05 ± 15.45 out of 80 (ranged 12−80), indicating a relatively moderate level of hypertension perception in patients.

In the MMAS‐8, most of patients answered “No” to the items 1 (*n* = 137, 51.7%), 4 (*n* = 138, 51.2%), 5 (*n* = 206, 77.7%), and 7 (*n* = 163, 61.5%), while most of them agreed that there were some days when they did not take their medicines (*n* = 176, 66.4%) and stopped taking their medication without telling their doctors, because they felt worse when they took it (*n* = 160, 60.4%) which were related to the items 2 and 3. Regarding the item 6 stating “When you feel like your condition is under control, do you sometimes stop taking your medicines?” there were almost equal percentage of answers (49.8% “yes” and 50.2% “no”). Finally, most of them (*n* = 165, 62.3%) reported that they “always” had difficulty remembering to take all their medications (item 8). The mean score of MMAS‐8 was obtained 3.69 ± 1.62 out of 8 (ranged 1−7), indicating that the treatment adherence in patients were at low level.

Results of Pearson's correlation test showed that the mean scores of total BIPQ‐R (*r* = 0.395, *p* < 0.001) and its eight components of illness consequences (*r* = 0.381, *p* < 0.001), illness duration (*r* = 0.238, *p* < 0.001), personal control (*r* = 0.362, *p* < 0.001), treatment control (*r* = 0.251, *p* = 0.004), symptoms (*r* = 0.398, *p* < 0.001), illness control (*r* = 0.307, *p* < 0.001), understanding (*r* = 0.264, *p* = 0.008), and emotional response (*r* = 0.296, *p* = 0.001), had a significant positive relationship with the MMAS‐8 score. Multiple regression analysis was conducted to find out which components of BIPQ‐R can predict treatment adherence in patients. The results showed that perceptions of illness consequences (*B* = 4.59, *p* = 0.005), personal control (*B* = 0.190, *p* = 0.047), and symptoms (*B* = 1.77, *p* = 0.005) could significantly predict treatment adherence (Table 2).

**Table 2:** Coefficients of regression analysis (dependent variable: treat adherence).

	Unstandardized coefficients		Standardized coefficients	*t*	Sig.	95% confidence interval	
	*B*	Standard error	Beta			Lower bound	Upper bound
(Constant)	10.446	0.955		10.938	0.000	8.565	12.327
Consequences	4.590	1.380	0.464	3.325	0.005	1.749	4.381
Duration	0.120	0.096	0.174	1.247	0.214	−0.069	0.308
Personal control	0.190	0.095	0.258	1.995	0.047	0.002	0.378
Treatment control	−0.141	0.089	−0.162	−1.582	0.115	−0.316	0.034
Symptoms	1.776	0.538	0.670	3.304	0.005	1.115	4.093
Concern	0.080	0.085	0.113	0.935	0.350	−0.088	0.248
Understanding	−0.112	0.085	−0.146	−1.323	0.187	−0.279	0.055
Emotional response	−0.039	0.082	−0.044	−0.481	0.631	−0.200	0.122

Results of the independent *t*‐test presented in Table 3 showed a significant difference in BIPQ‐R scores of patients in terms of place of residence (*p* = 0.032), where those living in urban areas had higher mean scores (48.25 ± 18.20) compared to those living in rural areas (42.66 ± 18.82). No significant difference was found in terms of gender (*p* = 0.791) and age (*p* = 0.231). Furthermore, results of one‐way ANOVA presented in Table 3 showed a significant difference in terms of education (*p* < 0.001) and occupation (*p* = 0.010) where those with university education and those who were employed had higher mean scores (*p* < 0.05). No significant difference was found in terms of marital status (*p* = 0.327).

**Table 3:** Mean scores of BIPQ‐R based on demographic factors, and the analysis results.

Characteristics		Mean ± SD	Independent *t*‐test results	One‐way ANOVA results
Gender	Male	46.67 ± 18.37	*T* = −0.265, *df* = 263, *p* = 0.790	‐
	Female	47.29 ± 18.54		
Marital status	Single	44.53 ± 19.15	^‐^	*F* = 1.121, *df* = 2, *p* = 0.327
	Married	48.08 ± 18.19		
	Divorced	48.65 ± 17.72		
Age (year)	≤40	44.81 ± 18.50	*T* = −1.570, *df* = 263, *p* = 0.230	‐
	>40	48.46 ± 18.33		
Educational level	Lower than high school	40.2 ± 18.09	‐	*F* = 10.784, *df* = 2, *p* < 0.001
	High school diploma	46.46 ± 18.29		
	Academic degree	52.52 ± 17.19		
Occupation	Unemployed	42.43 ± 19.24	‐	*F* = 3.825, *df* = 3, *p* = 0.010
	Employee	53.49 ± 14.19		
	Housekeeper	47.42 ± 18.59		
	Self‐employed	47.22 ± 18.95		
Place of residence	Urban areas	48.25 ± 18.20	*T* = 2.040, *df* = 263, *p* = 0.032	‐
	Rural areas	42.66 ± 18.82		

Abbreviations: BIPQ‐R, brief illness perception questionnaire‐revised; SD, standard deviation.

Results of independent *t*‐test presented in Table 4 showed a significant difference in MMAS‐8 scores of patients in terms of place of residence (*p* = 0.042) where those living in urban areas had slightly higher adherence to treatment (2.92 ± 1.61) compared to those living in rural areas (2.76 ± 1.65). No significant difference was found in terms of gender (*p* = 0.357) and age (*p* = 0.376). Moreover, results of one‐way ANOVA presented in Table 4 showed a significant difference in terms of education (*p* = 0.045), where those with university education had higher adherence compared to other education groups (*p* < 0.05). No significant difference was found in terms of marital status (*p* = 0.260) and occupation (*p* = 0.719).

**Table 4:** Mean scores of MMAS‐8 based on demographic factors, and the analysis results.

Characteristics		Mean ± SD	Independent *t*‐test results	One‐way ANOVA results
Gender	Male	2.61 ± 1.66	*T* = −0.923, *df* = 263, *p* = 0.357	‐
	Female	2.90 ± 1.60		
Marital status	Single	12.47 ± 1.98	‐	*F* = 1.352, *df* = 2, *p* = 0.260
	Married	12.76 ± 2.11		
	Divorced	13.18 ± 2.45		
Age (year)	≤40	2.64 ± 1.59	*T* = −0.886, *df* = 263, *p* = 0.376	‐
	>40	2.83 ± 1.64		
Educational level	Lower than high school	2.97 ± 1.59	‐	*F* = 2.337, *df* = 2, *p* = 0.045
	High school diploma	3.21 ± 1.73		
	Academic degree	3.47 ± 1.54		
Occupation	Unemployed	2.66 ± 1.52	‐	*F* = 0.448, *df* = 3, *p* = 0.719
	Employee	2.80 ± 1.62		
	Housekeeper	2.92 ± 1.66		
	Self‐employed	2.73 ± 1.71		
Place of residence	Urban areas	2.92 ± 1.61	*T* = 1.362, *df* = 263, *p* = 0.042	‐
	Rural areas	2.76 ± 1.65		

Abbreviations: MMAS, Morisky medication adherence scale; SD, standard deviation.

## 4 DISCUSSION

The purpose of this study was to investigate hypertension treatment adherence and perception of hypertension in hypertensive patients attending a teaching hospital in western Iran. The results showed a moderate level of illness perception (mean BIPQ‐R score = 49.05 ± 15.45) and a low level of treatment adherence (mean MMAS‐8 score = 3.69 ± 1.62). In Taheri‐Kharameh et al.'s study in 2016^21^ in northern Iran (Qom City) on 140 hypertensive patients, about 35% showed higher adherence to treatment based on the score of the Hill‐Bone Compliance to High Blood Pressure Therapy Scale. In Zare et al.'s study in 2018 on adherence of patients to antihypertensive drug use in Shiraz City,^31^ it was reported that most of patients had low adherence (76.95%) which is consistent with our result. In the study by Doust Mohammadi et al. in Tehran City in 2018 and Bagheri et al. in 2019 in Urmia City (north and northwest of Iran), the mean MMAS‐8 score of patients was 6.9 ± 1.5 and 5.21 ± 2.27, respectively^22^, ^32^ which are higher than the scores reported in our study. This discrepancy may be related to the difference in the study area (Western Iran vs. northern Iran). Saarti et al.^20^ in a study on Lebanese hypertensive patients, found that 29.1% of 117 patients had poor adherence to antihypertension treatment based on the MMAS‐8 score. In Guimarães et al.'s study^23^ in Portugal, hypertensive patients reported a high level of medication adherence. In Otenyo and Kereri's study^24^ in Kenya. 33.3% of the hypertensive patients had a high adherence level. These fining are not consistent with our results. In Al‐Ramahi's study^33^ on Palestinian hypertensive patients, low adherence with medications was present in 244 (54.2%) of the patients. In Rashid et al.' study,^25^ compliance to antihypertensive medication among hypertensive patients in Dhaka Medical College Hospital in Bangladesh was very low (about 15%).

According to the perceptions of the most of patients in our study, the hypertension had moderately affected their life and emotions, and they had a moderate amount of control over the disease; treatment was moderately helpful for them; they were moderately concerned about their illness, and understood their illness moderately. In terms of adherence to treatment, most of patients in the present study reported that they remember to take medications and bring along their medications when travel or leave home; and were not feeling hassled or tired about sticking to their treatment plan. However, there were some days when they did not take their medicines and stopped taking their medication without telling their doctors, because they felt worse when they took it. They always had difficulty remembering to take all their medications. We found a significant relationship treatment adherence and perception of illness in patients. This is consistent with the findings of Taheri‐Kharameh et al. and Doust Mohammadi et al. in Iran, Shakya et al. in Nepal, Guimarães et al. in Portugal, and Otenyo and Kereri in Kenya.^18^, ^21^, ^22^, ^23^, ^24^ In Saarti et al.'s study in Lebanon,^20^ although the mean BIPQ score of adherent participants was lower than the mean score of nonadherent participants, this difference was not statistically significant which is against our results. In Eke's study in 2018, only the BIPQ domain of “consequences” had a statistically significant relationship with medication adherence.^34^ Chen et al. in a study in 2011 on hypertensive patients in Taiwan, reported that the illness perception may directly affect medication adherence in patients or indirectly affect their adherence by controlling the disease.^35^

This is the first study that measures both treatment adherence and illness perceptions in hypertensive patients in western Iran. However, there were some limitations/disadvantages in this study including difficulty in recruiting patients due to the COVID‐19 pandemic, the uptake of many medications by patients which can reduce their adherence, and the use of self‐report tools which may provide biased responses. Other disadvantage was the lack of examining the polypharmacy of antihypertensive medications. Moreover, caution should be made in generalizing the results to all hypertensive patients in Iran. Further studies are recommended for assessing treatment adherence and illness perceptions in hypertensive patients living in the east and south of Iran. More studies in Iran are needed to determine which interventions can be used to improve treatment adherence and illness perception of hypertensive patients. Qualitative studies are needed to better understand the causes of nonadherence to hypertension treatment among patients in the study area and find solutions to increase their adherence. The hypertensive patients should be informed about the consequences of nonadherence to treatment regimen through social media or TV programs.

## 5 CONCLUSION

Treatment adherence of hypertensive patients in western Iran is at low level, but their perception of hypertension is at moderate level. It is necessary for clinical physicians to pay attention to the perceptions of these patients about consequences, personal control, and symptoms of hypertension to improve their adherence to hypertension treatment.

## AUTHOR CONTRIBUTIONS

**Parastoo Baharvand**: Software; supervision; validation. **Farideh Malekshahi**: Project administration; supervision; writing—review and editing. **Amirpourya Babakhani**: Conceptualization; data curation; formal analysis; investigation; writing—original draft.

## CONFLICT OF INTEREST STATEMENT

The authors declare no conflict of interest.

## ETHICS STATEMENT

All procedures performed in this study were in accordance with the ethical standards of the ethics committee of Lorestan Univeesity of Medical Sciences and with the 1964 Helsinki declaration. The ethical approval was obtained the Research Ethics Committee of Lorestan University of Medical Sciences (Code: IR.LUMS.REC.1399.223). Informed consent was obtained from all individual participants included in the study.

## TRANSPARENCY STATEMENT

The lead author Farideh Malekshahi affirms that this manuscript is an honest, accurate, and transparent account of the study being reported; that no important aspects of the study have been omitted; and that any discrepancies from the study as planned (and, if relevant, registered) have been explained.

## Data Availability

The data sets used and analyzed during the current study are available from the corresponding author on reasonable request.

## References

[1] Kjeldsen SE . Hypertension and cardiovascular risk: general aspects. Pharmacol Res. 2018;129:95‐99. 29127059 10.1016/j.phrs.2017.11.003

[2] Mirzaei M , Mirzaei M , Bagheri B , Dehghani A . Awareness, treatment, and control of hypertension and related factors in adult Iranian population. BMC Public Health. 2020;20(1):667. 32397990 10.1186/s12889-020-08831-1 PMC7216488

[3] Hypertension WHO . Hypertension 2021. https://www.who.int/news-room/fact-sheets/detail/hypertension.

[4] Oori MJ , Mohammadi F , Norozi K , Fallahi‐Khoshknab M , Ebadi A , Gheshlagh RG . Prevalence of HTN in Iran: meta‐analysis of published studies in 2004‐2018. Current Hypertension Rev. 2019;15(2):113‐122. 10.2174/1573402115666190118142818 PMC6635676 30657043

[5] Esteghamati A , Abbasi M , Alikhani S , et al. Prevalence, awareness, treatment, and risk factors associated with hypertension in the Iranian population: the national survey of risk factors for noncommunicable diseases of Iran. Am J Hypertens. 2008;21(6):620‐626. 18451810 10.1038/ajh.2008.154

[6] Kearney PM , Whelton M , Reynolds K , Muntner P , Whelton PK , He J . Global burden of hypertension: analysis of worldwide data. Lancet. 2005;365(9455):217‐223. 15652604 10.1016/S0140-6736(05)17741-1

[7] Mills KT , Bundy JD , Kelly TN , et al. Global disparities of hypertension prevalence and control: a systematic analysis of population‐based studies from 90 countries. Circulation. 2016;134(6):441‐450. 27502908 10.1161/CIRCULATIONAHA.115.018912 PMC4979614

[8] Mancia G , Fagard R , Narkiewicz K , et al. 2013 ESH/ESC guidelines for the management of arterial hypertension: the task force for the management of arterial hypertension of the European Society of Hypertension (ESH) and of the European Society of Cardiology (ESC). J Hypertens. 2013;31(7):1281‐1357. 23817082 10.1097/01.hjh.0000431740.32696.cc

[9] Mahmood S , Shah KU , Khan TM , et al. Non‐pharmacological management of hypertension: in the light of current research. Irish J Med Sci. 2019;188(2):437‐452. 30136222 10.1007/s11845-018-1889-8

[10] Jordan J , Kurschat C , Reuter H . Arterial hypertension. Dtsch Arztebl Int. 2018;115(33‐34):557‐568. 30189978 10.3238/arztebl.2018.0557 PMC6156553

[11] Manolis AJ , Poulimenos LE , Kallistratos MS . Arterial hypertension: benefits and limitations of treatment 2015, 13(28). https://www.escardio.org/Journals/E-Journal-of-Cardiology-Practice/Volume-13/arterial-hypertension-benefits-and-limitations-of-treatment

[12] Bailey JE , Wan JY , Tang J , Ghani MA , Cushman WC . Antihypertensive medication adherence, ambulatory visits, and risk of stroke and death. J Gen Intern Med. 2010;25(6):495‐503. 20165989 10.1007/s11606-009-1240-1 PMC2869423

[13] Mazzaglia G , Ambrosioni E , Alacqua M , et al. Adherence to antihypertensive medications and cardiovascular morbidity among newly diagnosed hypertensive patients. Circulation. 2009;120(16):1598‐1605. 19805653 10.1161/CIRCULATIONAHA.108.830299

[14] Corrao G , Parodi A , Nicotra F , et al. Better compliance to antihypertensive medications reduces cardiovascular risk. J Hypertens. 2011;29(3):610‐618. 21157368 10.1097/HJH.0b013e328342ca97

[15] World Health Organization . Adherence to Long‐term Therapies: evidence for Action. World Health Organization; 2003.

[16] Ross S , Walker A , MacLeod MJ . Patient compliance in hypertension: role of illness perceptions and treatment beliefs. J Hum Hypertens. 2004;18(9):607‐613. 15029218 10.1038/sj.jhh.1001721

[17] Tan CS , Hassali MA , Neoh CF , Saleem F . A qualitative exploration of hypertensive patients' perception towards quality use of medication and hypertension management at the community level. Pharm Pract. 2017;15(4):1074. 10.18549/PharmPract.2017.04.1074 PMC5742001 29317924

[18] Shakya R , Shrestha S , Gautam R , et al. Perceived illness and treatment adherence to hypertension among patients attending a tertiary hospital in Kathmandu, Nepal. Patient Prefer Adherence. 2020;14:2287‐2300. 33244224 10.2147/PPA.S270786 PMC7685346

[19] Hsiao CY , Chang C , Chen CD . An investigation on illness perception and adherence among hypertensive patients. Kaohsiung J Med Sci. 2012;28(8):442‐447. 22892166 10.1016/j.kjms.2012.02.015 PMC11916609

[20] Saarti S , Hajj A , Karam L , et al. Association between adherence, treatment satisfaction and illness perception in hypertensive patients. J Hum Hypertens. 2016;30(5):341‐345. 26310182 10.1038/jhh.2015.86

[21] Taheri‐Kharameh Z , Hazavehei SMM , Ramezani T , Vahedi A , Khoshro M , Sharififard F . The assessment of illness perception and adherence to therapeutic regimens among patients with hypertension. J Educ Commun Health. 2017;3(2):9‐15.

[22] Doust Mohammadi S , Norouzi K , Dalvandi A , Norouzi M . The level of illness perception and its relationship with adherence to the medical regimen in the elderly with hypertension. Iranian J Rehabil Res Nurs. 2018;4(3):40‐46.

[23] Guimarães TCA , Graça A , Silva AM , Fonseca AR . Illness perceptions and medication adherence in hypertension. BMC Health Serv Res. 2016;16:O135.

[24] Otenyo SOKD . Illness perceptions and adherence to medication regimen among hypertensive patients attending a county referral hospital in Kenya. J Hypertens Manag. 2021;7:059.

[25] Rashid MM , Haque MM , Islam MS , Parvin MF , Kahhar A . Noncompliance to antihypertensive medication in patients with essential hypertension attending Dhaka Medical College Hospital. TAJ: J Teachers Associa. 2023;35(2):103‐110.

[26] Broadbent E , Petrie KJ , Main J , Weinman J . The brief illness perception questionnaire. J Psychosom Res. 2006;60(6):631‐637. 16731240 10.1016/j.jpsychores.2005.10.020

[27] Masaeli NBR , Kheirabadi G , Khedri A , Mahaki B . Psychometric properties of the Persian version of the brief illness perception questionnaire in chronic diseases. J Rehabil Sci. 2019;14(16):332‐337.

[28] Morisky DE , Green LW , Levine DM . Concurrent and predictive validity of a self‐reported measure of medication adherence. Med Care. 1986;24(1):67‐74. 3945130 10.1097/00005650-198601000-00007

[29] Morisky DE , Ang A , Krousel‐Wood M , Ward HJ . Predictive validity of a medication adherence measure in an outpatient setting. J Clin Hypertension. 2008;10(5):348‐354. 10.1111/j.1751-7176.2008.07572.x PMC2562622 18453793

[30] Moharamzad Y , Saadat H , Nakhjavan Shahraki B , et al. Validation of the Persian version of the 8‐item morisky medication adherence scale (MMAS‐8) in Iranian hypertensive patients. Glob J Health Sci. 2015;7(4):173‐183. 25946926 10.5539/gjhs.v7n4p173 PMC4802120

[31] Zare S , Shams M , Fararouei M , Shariatinia S . Antihypertensive drugs adherence in heart disease patients referring to the Imam Reza Clinic in Shiraz. Sadra Med J. 2018;6(2):151‐160.

[32] Bagheri R , Masudi S , Salarilak S , Khademvatani K , Khalkhali HR . Adherence to hypertension treatment and its determinants in patients referred to a tertiary cardiology center in Urmia, Iran. Avicenna J Clin Med. 2019;26(2):83‐92.

[33] Al‐Ramahi R . Adherence to medications and associated factors: a cross‐sectional study among Palestinian hypertensive patients. J Epidemiol Global Health. 2015;5(2):125‐132. 10.1016/j.jegh.2014.05.005 PMC7320483 25922321

[34] Eke S . Association of illness perception and antihypertensive medication adherence among black hypertensive adults in primary care settings: the University of Texas. 2018.

[35] Chen SL , Tsai JC , Chou KR . Illness perceptions and adherence to therapeutic regimens among patients with hypertension: a structural modeling approach. Int J Nurs Stud. 2011;48(2):235‐245. 20678768 10.1016/j.ijnurstu.2010.07.005

